# Neutrophil Dynamics Affect *Mycobacterium tuberculosis* Granuloma Outcomes and Dissemination

**DOI:** 10.3389/fimmu.2021.712457

**Published:** 2021-10-05

**Authors:** Caitlin Hult, Joshua T. Mattila, Hannah P. Gideon, Jennifer J. Linderman, Denise E. Kirschner

**Affiliations:** ^1^ Department of Mathematics, Gettysburg College, Gettysburg, PA, United States; ^2^ Department of Infectious Diseases and Microbiology, University of Pittsburgh School of Public Health, Pittsburgh, PA, United States; ^3^ Center for Vaccine Research, University of Pittsburgh, Pittsburgh, PA, United States; ^4^ Department of Microbiology and Molecular Genetics, University of Pittsburgh School of Medicine, Pittsburgh, PA, United States; ^5^ Department of Chemical Engineering, University of Michigan, Ann Arbor, MI, United States; ^6^ Department of Microbiology and Immunology, University of Michigan Medical School, Ann Arbor, MI, United States

**Keywords:** tuberculosis (TB), dissemination, nonhuman primate (NHP), sensitivity analysis (SA), agent-based model (ABM), neutrophil

## Abstract

Neutrophil infiltration into tuberculous granulomas is often associated with higher bacteria loads and severe disease but the basis for this relationship is not well understood. To better elucidate the connection between neutrophils and pathology in primate systems, we paired data from experimental studies with our next generation computational model *GranSim* to identify neutrophil-related factors, including neutrophil recruitment, lifespan, and intracellular bacteria numbers, that drive granuloma-level outcomes. We predict mechanisms underlying spatial organization of neutrophils within granulomas and identify how neutrophils contribute to granuloma dissemination. We also performed virtual deletion and depletion of neutrophils within granulomas and found that neutrophils play a nuanced role in determining granuloma outcome, promoting uncontrolled bacterial growth in some and working to contain bacterial growth in others. Here, we present three key results: We show that neutrophils can facilitate local dissemination of granulomas and thereby enable the spread of infection. We suggest that neutrophils influence CFU burden during both innate and adaptive immune responses, implying that they may be targets for therapeutic interventions during later stages of infection. Further, through the use of uncertainty and sensitivity analyses, we predict which neutrophil processes drive granuloma severity and structure.

## 1 Introduction

Infection with *M. tuberculosis* (Mtb), the bacterium that causes tuberculosis (TB), induces immune responses that culminate in the formation of multicellular lesions called granulomas. This response is highly effective at restricting bacterial replication and most individuals never experience active (symptomatic) disease. Approximately one-quarter to one-third of the world’s population is infected with Mtb and is susceptible to developing or progressing to active TB (1). Until the recent COVID-19 pandemic, TB remained the world’s deadliest infectious disease, with 1.5 million or more people dying per year (1, 2). The global burden of Mtb infection, coupled with increasing rates of drug resistance, emphasize the urgent need to identify factors that contribute to protective and pathologic outcomes in TB.

Neutrophils are innate immune cells whose role in TB remains poorly understood. Numerous, short-lived, and easily activated, neutrophils are phagocytic cells that are typically associated with acute inflammatory responses (3–5). These characteristics make them difficult to study experimentally, particularly within the context of a disease that progresses slowly and can remain undetected for years. In contrast to their role in effectively killing bacteria and reducing bacterial loads in other infectious diseases (6), neutrophils appear to have an impaired capacity for killing phagocytosed Mtb (5). This suggests that neutrophils may promote TB by providing Mtb with a temporary intracellular niche for survival and replication (4, 7). Similarly, by engulfing Mtb but not killing them, highly-motile neutrophils may contribute to dissemination by providing mycobacteria a means of moving within the lung environment (5, 8–10).

A search of the literature on the role of neutrophils in TB reveals a lack of broad scientific consensus on whether they contribute to protection or drive pathologic outcomes. Some of the ambiguity may be attributed to different pathologic presentation and immune function between murine TB models and human TB (11), stage of disease at which granulomas can be harvested from non-human primates (NHPs) with TB, and paucity of studies on human granulomas that represent different stages of disease. That said, the available data highlight the potential importance of the stage of infection and the spatial location of neutrophils within a granuloma for determining neutrophil-related outcomes (5, 8, 12–15). Recent experimental work has shown that more neutrophils per granuloma correlate with higher levels of inflammation and poorer host outcomes, suggesting a link between neutrophils and progression to advanced disease (4, 12, 16, 17). However, other work suggests a more complicated picture (5, 18, 19). Finally, it is likely that a balance of pro- and anti-inflammatory molecules works to maintain long term control of infection during chronic infections (20). It could be that neutrophils, which can produce both pro- and anti-inflammatory cytokines (21), may tip the granuloma environment toward a more bacteria-permissive space.

That primate Mtb infection results in multiple lung granulomas with their own independent trajectories complicates issues further (22, 23). At the host level, there can be substantial heterogeneity among granuloma outcomes where some granulomas in Mtb-infected NHPs experience uncontrolled bacteria growth and inflammation while other granulomas develop sterilizing immunity and kill all the bacteria they contain (22). There is evidence that the failure of only one granuloma to contain bacterial growth can be sufficient to lead to a poor outcome for the host (24). Neutrophils may contribute to these outcomes but have been difficult to study in this model because neutrophil depletion studies cannot be performed in NHPs. Moreover, granuloma formation during the innate immune response may be important for disease outcome, but it is difficult to identify such granulomas using positron emission tomography/computed tomography (PET/CT) or visual inspection at necropsy. Thus, we know little about neutrophil biology in the earliest stages of NHP or human TB and our understanding of neutrophil biology in later stages of TB is likely biased by studies investigating severe pathology and poorly controlled disease.

Computational models of granuloma formation and function represent a complementary tool for identifying neutrophil-regulated processes that differentiate protective and pathologic granulomas (25). While experimental work is confined to the biological timescales of TB progression (months to years), computational modelers can conduct simulated experiments with runtimes on the order of minutes to hours. Here, we build on our multiscale and mechanistic computational model of granulomas, *GranSim*, to identify the contributions of neutrophils to immunity at the granuloma scale. *GranSim* has been continually curated for 15 years and captures numerous aspects of granuloma biology including T cell and macrophage behaviors, Mtb fate, and granuloma-tissue scale outcomes (26–31). To date, neutrophils have not been extensively modeled in *GranSim*, as they have been grouped in with other phagocytic cells rather than distinguished as their own cell type. In order to explore the distinct contributions of neutrophils, we follow the lead of Bru and Cardona (2010), who introduced a computational model that included explicit simulation of neutrophils within the broader immune response to infection with Mtb (32). Among other key findings, they showed that local chemokine concentrations and especially the adaptive immune response affect granuloma formation and Mtb levels, and offered an explanation for why granulomas cannot be visualized in the first weeks post-infection. In *GranSim*, we significantly expand and fine-grain the range of neutrophil behaviors and parameters presented in (32); through simulating neutrophil behaviors (e.g., secretion, phagocytosis/transport/killing of Mtb), defining additional neutrophil parameters (e.g., chemotactic/recruitment factors), and allowing variation of parameters (e.g., neutrophil lifespan), we capture a broad picture of neutrophil function in *GranSim* and can now address mechanistic questions regarding the role of neutrophils in granuloma outcome.

In this study, we use a systems biology approach. We present a comprehensive, neutrophil-inclusive update to *GranSim* that significantly advances the integration of neutrophil behaviors into the granuloma environment. We pair this with studies on NHP granulomas to calibrate and validate the model. We are interested in determining the mechanisms that drive granuloma severity and structure and in predicting the relevance of neutrophils to the development of new therapeutic strategies for TB. Using this model, we investigate which processes influence CFU burden and neutrophil count, whether neutrophil behavior during the adaptive immune response affects CFU burden, and whether neutrophil mechanisms drive phenomena such as bacterial dissemination.

## 2 Materials and Methods

### 2.1 Animal Ethics Statement

The tissue used to generate the flow cytometry dataset and IHC imaging used in this study originated from cynomolgus macaques (*Macaca fascicularis*) that were enrolled in studies at the University of Pittsburgh. The studies these animals were involved in were approved by the University of Pittsburgh’s Institutional Animal Care and Use Committee (IACUC) and were performed in BSL3 facilities approved by Environmental Health and Safety at the University of Pittsburgh. Animals were infected with Mtb (Erdman strain) as previously performed (33) and the animals were housed under BSL3 conditions and housed and maintained in accordance with standards established in the Animal Welfare Act and the Guide for the Care and Use of Laboratory Animals. The University of Pittsburgh is an American Association of Laboratory Animal Sciences (AAALAS) certified program. At the end of the study, animals were humanely euthanized and necropsied as previously described (33).

### 2.2 Non-Human Primate Studies

The macrophage and T cell count data used in this study were derived from a dataset composed of 30 granulomas from 7 Mtb-infected NHPs presented in Wessler et al. (2020) (34). Neutrophil cell count data were derived from the same dataset and are used for the first time in this work. All cell count data were extracted from granulomas in cynomolgus macaques that were infected with Mtb for 3, 5, 7, or 9 weeks (34). Briefly, the individual granulomas were excited from macaques and enzymatically digested using a gentle MACS tissue dissociator. The single cell suspension obtained by enzymatic digestion was processed for bacterial burden and cell numbers enumeration (35). Single cell suspensions of granuloma cells were stained with cell surface antibodies to enumerate T cells (CD3) and myeloid cells (CD11b+). The cells were further stained intracellularly with calprotectin antibody to define neutrophil (CD11b+calprotectin+) and macrophage (CD11b+calprotectin-) populations. Flow cytometry and data acquisition were performed using a BD LSRII and analysis was performed using Flowjo Software v10 (35). The CFU dataset used to calibrate this model is composed of 623 granulomas from 38 NHPs (previously published (34) and ongoing studies). Whereas macrophage and T cell count datasets were used in model calibration, neutrophil cell count datasets were primarily used to validate our model predictions.

### 2.3 Modeling the Immune Response to Mtb Using a Hybrid Agent-Based Computational Model, GranSim

We use our 2D hybrid agent-based multi-scale model of the immune response to Mtb infection. We present a neutrophil-inclusive computational model that significantly updates our existing hybrid agent-based computational model, known as *GranSim* (granuloma simulator) (26–31). Continuously curated with experimental datasets since its inception, *GranSim* simulates the spatiotemporal dynamic formation of primate lung granulomas during the days, months, and even years following Mtb infection. It captures immune processes over molecular, cellular, and tissue scales to simulate the movement of thousands of cells and bacteria, cytokine and chemokine dynamics, and intracellular and extracellular processes. *GranSim* defines the following as distinct cell types: macrophages (further distinguished by state as resting, activated, infected, or chronically infected), T cells (further distinguished by type as cytotoxic, regulatory, and IFN*_Ɣ_* producing), and now neutrophils. *GranSim* simulates concentration gradients of cytokines and chemokines, including TNF-*α*, IL10, CCL2, CCL5, CXCL-9, and TGF-*ß*. We also track dead tissue, quantifying caseum levels throughout the granuloma environment. Finally, each bacterium is tracked individually [as if uniquely barcoded (22, 36, 37)], and we distinguish among bacteria in different environments: as extracellular (replicating and non-replicating), intracellular (macrophages), or intracellular (neutrophils). We scale our 2D simulated data to 3D (e.g., 3D CFU) using the scaling factors and methods established in Renardy et al. (2019) (38).

As an agent-based model (ABM), *GranSim* consists of four primary components, or building blocks: a grid, where each grid compartment is 20 um x 20 um in size, while the entire grid is 4 mm x 4 mm in size; the timescales over which events occur (e.g., seconds to years); the agents (e.g., macrophages, T cells, neutrophils, bacteria); and the rules that describe how those agents behave and interact on the grid (e.g., cell speed, secretion, chemotaxis properties). Our ABM enables us to capture both the stochasticity observed in biological systems and the marked distinctions in behavior among different cell types. Granuloma formation within this model is an emergent behavior rather than a prescribed model outcome. A full list of model rules prior to adding in neutrophils and an executable file are provided at the *GranSim* website: http://malthus.micro.med.umich.edu/GranSim. Describing immune processes over molecular, cellular, and tissue scales, *GranSim* is a multi-scale hybrid model whose model scales are linked to one another through cytokine concentrations and/or agent behaviors, as has been described previously (39).

### 2.4 Incorporating Neutrophils as a Cell in GranSim

**Figure 1:**
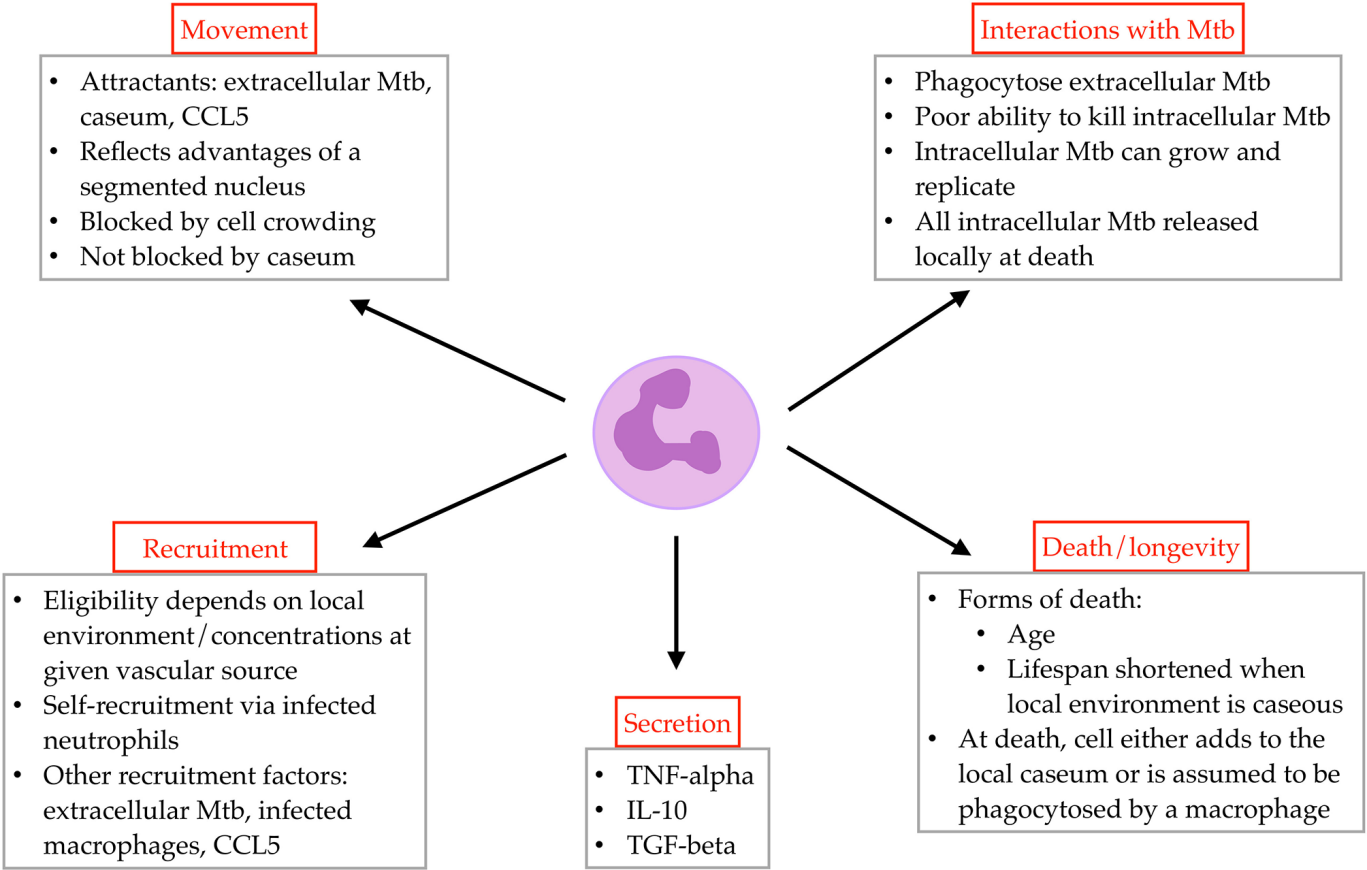
Overview of rules governing neutrophil agent behavior in *GranSim*.

We include neutrophils as a unique agent class in *GranSim*, alongside the existing agent classes for macrophages, T cells, and bacteria. Below we elaborate the biological rules and assumptions that are included in *GranSim* (e.g., interactions with other agents or chemical gradients, requirements for recruitment to the grid). We also identify neutrophil-specific parameters including neutrophil lifespan, phagocytosis of extracellular Mtb, and chemotaxis (described below; **Figure 1** and **Online Supplement Figure 1**).

#### 2.4.1 Model Initialization

Although neutrophils are present in the lung during normal health conditions (40, 41), they are recruited to the lung from the blood following infection (6, 42). Due to the short lifespan of neutrophils, the abundance of both blood vessels in the lung and neutrophils in the blood, and the lack of *in vivo* systems where neutrophil recruitment in early disease can be assessed, we do not initialize the virtual healthy lung tissue with any neutrophils but instead allow them to be rapidly recruited after infection. Through effectively allowing neutrophil recruitment from the blood to dictate neutrophil numbers in the virtual lung, we achieve biologically-accurate neutrophil numbers in our simulated granulomas.

#### 2.4.2 Neutrophil Lifespan, Size, and Death

Reported values for neutrophil lifespan vary widely, ranging from 5 hours to 5.4 days in the circulation (15, 43–46). Furthermore, various biological factors influence neutrophil lifespan, including their global location (e.g., in the bloodstream versus the tissues) and their local location (e.g., in the highly stimulatory environment of a granuloma) (3, 45). Therefore, we define neutrophil lifespan using a range rather than a single value to better capture this variability.

Typically, 12-14 um in size, neutrophils are easily recognizable by their segmented nuclei, which enables them to migrate through crowded regions of the lung environment more effectively than other cells. In *GranSim*, we simulate neutrophils as a “squeezing” cell, capable of moving or being recruited into grid compartments that already contain a T cell, macrophage, or another neutrophil.

The neutrophil lifespan is short, especially in comparison to those of other cell types in the model. Once a neutrophil dies, it contributes to caseum in its vicinity, or is cleared *via* phagocytosis (efferocytosis) by a macrophage (3, 13, 40); we capture this behavior through two probability parameters. For neutrophils that may have phagocytosed Mtb, any intracellular Mtb are released into the local environment upon the neutrophil’s death.

#### 2.4.3 Neutrophil Movement, Recruitment, and Cytokine and Chemokine Production

In the model, neutrophils move at the same speed as T cells, at a rate of 2 um/minute (47, 48) and once every agent time step. Directional movement is driven by chemotactic factors in the lung environment. The local environment translates to the Moore neighborhood (MN) of a given cell, and the composite, weighted concentration of neutrophilic chemotactic factors in the MN dictates the grid compartment to which a neutrophil will move. Neutrophil chemotactic factors in *GranSim* include local levels of extracellular Mtb, caseum, and CCL5 (8, 9, 49, 50).

Neutrophils secrete an array of cytokines and chemokines during Mtb infection (8, 21, 51–54). In *GranSim*, neutrophils contribute to the pro-inflammatory environment through secretion of TNF-*α* (8, 21, 55, 56), and to the anti-inflammatory environment through secretion of IL-10 (8, 14, 21, 57) and TGF-β (58, 59). Neutrophils express significant amounts of IL-8 and thus likely help drive their own recruitment; we account for the effects of neutrophil secretion of IL-8 through the inclusion of infected neutrophil presence as a recruitment factor for neutrophils. The ability and impact of neutrophil cytokine secretion remains an area of active investigation.

We assume that recruitment of neutrophils to the lung is determined by the cumulative, weighted concentrations of extracellular Mtb, intracellular Mtb within macrophages, infected neutrophils, and CCL5 near a given vascular source (3, 14, 40, 50, 60). The inclusion of infected neutrophils as a recruitment factor is a proxy for neutrophil secreted factors that increase neutrophil recruitment. (Future models might include additional molecular factors.) A caseum-occluded vascular source cannot admit neutrophils and is considered a “blocked” compartment for all cell recruitment.

#### 2.4.4 Neutrophil Interactions With Mtb and Caseum

Neutrophils phagocytose Mtb during the course of infection (13, 61). Researchers have investigated the proportion of Mtb-infected cells that are neutrophils and how that proportion changes over time (5, 7, 62). In *GranSim*, we allow neutrophils to phagocytose extracellular Mtb using parameters defining the number of bacteria that a neutrophil can take up at a given time point and the maximum number of intracellular bacteria allowed in a neutrophil. Intracellular bacteria can replicate rapidly in neutrophils (7). Although Eum et al. (2010) present airway-level rather than granuloma-level data, this work is an important example of native Mtb in human neutrophils and thus provides a starting point for neutrophil Mtb growth kinetics in our model. Neutrophil anti-mycobacterial activity is thought to be largely ineffective (4, 9), and variable (5); thus, we define the probability that a neutrophil kills its intracellular bacteria as a parameter that varies across a range of values, in order to capture these observed biological variations and uncertainty.

Although neutrophils can enter caseous regions (21, 53, 63), this microenvironment is hypoxic (64) and accelerates cell death. We do not prevent neutrophils from moving into fully-caseasted compartments; however, we reduce their remaining lifespans for each timestep that they are in a caseated compartment.

### 2.5 Model Calibration of GranSim

We calibrated the model to experimental datasets that are different from those used to validate the model. This was a multistep process that involved comparison to both temporal and spatial data, as well as phenomenological behavior. We used two primary types of data: i) temporal CFU data, ii) temporal T cell/macrophage ratio data. We also required formation of a spatial structure characteristic of a necrotic granuloma at specific points in time. Additionally, as further controls, we performed simulated virtual TNF and T cell deletions to ensure that model behavior recapitulates the known *in vivo* behavior, as we have done previously (results not shown). We assigned values (specific or ranges) to new model parameters based on the following: experimental datasets from our collaborators, data from the literature, and, in the case of “proxy’’ parameters, values derived from past work with *GranSim*. The parameter **Online Supplement Table 1** indicates how values were determined.

#### 2.5.1 Uncertainty and Sensitivity Analyses

In order to identify neutrophil mechanistic parameters and not bias the model toward limited datasets, we assigned broad ranges to parameter values. This uncertainty analysis allows us to explore a full range of behaviors around starting values provided in literature. We then narrow the parameter ranges as we validate to both spatial and temporal neutrophil datasets. Specifically, we perform uncertainty and sensitivity of the model to determine the biologically relevant parameter space and identify parameters that are significantly correlated with granuloma outcomes. We used Latin Hypercube Sampling (LHS) to efficiently sample the entire parameter space, using previously developed methodology (65, 66). In order to perform simulations with a range of parameters, it becomes necessary to capture both aleatory and epistemic uncertainty (67). Epistemic uncertainty is captured by the variation in parameters (ranges); however, aleatory uncertainty is captured by the stochastic nature of the simulations. Thus 3-5 repetitions are performed on each run to observe how changes in both probabilities and parameter values affect model outputs.

To correlate which parameters induced variability in outcomes, we perform sensitivity analysis. To do this, we rank the correlations (and therefore, contributions) of parameters to important outcome statistics like CFU. We use Partial Rank Correlation Coefficients by identifying coefficients of ranked correlations (PRCC), which are designed to study non-linear system correlations. PRCC is a tool for analyzing which model parameters drive different model outputs, and we use it here according to how we have previously (65). PRCC values are significant if they satisfy a p-value; here we use p < 0.001. PRCC values range from -1 to 1, with negative values indicating a negative correlation between a given parameter and output statistic and positive values indicating a positive correlation between that parameter and statistic. Values of higher magnitudes indicate stronger relationships between parameters and statistics, but a Fishers Z Test must be performed in order to directly compare parameters and determine if they are statistically different from each other (65, 68, 69).

When significance is tested for multiple parameters, then *p*-value corrections are needed to reduce type I error (70). Corrections are needed when performing multiple significance tests because more tests lead to more false positives. For example, a significance level of *α*=0.05 indicates that there is a 5% probability of a false positive for a single significance test. For multiple tests, the probability of a false positive for at least one test is 1-0.95*^n^*, where *n* is the number of tests performed. Thus, if one is performing 10 tests, there is a 40% chance that at least one test will result in a false positive. To address this issue, there are several correction methods available (71). Bonferroni correction controls the family-wise error rate, where raw *p*-values are multiplied by the number of tests (or, equivalently, the significance level is divided by the number of tests). This ensures that the family-wise error rate, i.e., the probability of at least one false positive, is no more than *α*; equivalently, this tests the composite null hypothesis that all correlations are zero. In the FDR method, *p*-values are ranked in ascending order and multiplied by *n/k* where *k* is the rank of a *p*-value and *n* is the number of tests. This ensures that if a relationship is deemed significant, the probability that it is a false positive is no more than α. Here when we applied it to our sensitivity analysis (see *Results*) we reduced the number of significant parameters by seven.

#### 2.5.2 Steps in the Calibration Pipeline

We calibrated the model to datasets derived from Mtb-infected NHPs. We use LHS to efficiently sample the parameter space, as described above. Through varying 77 total parameters, 17 of which directly describe neutrophil behavior, we explored a large section of parameter space and diverse model outcomes. We iteratively reduced this parameter space through calibration to experimental data; namely, we discarded those simulated granulomas that did not sufficiently capture experimental temporal trends in total CFU and T cell to macrophage cell count ratio, before reapplying this sampling method to the newly reduced parameter space. In **Online Supplement Table 1**, we present the final full parameter set, in which we simulated 600 granulomas 3 times each. Using this parameter set, we show that our model produces a range of biologically-realistic granulomas. This is a positive qualitative feature of the model. As the model should capture a range of experimental outcomes on both temporal and spatial scales, we note that our parameter space remains relatively large and includes a wide range of outcomes including resolving granulomas that develop sterilizing immunity and progressing granulomas with uncontrolled bacterial replication and dissemination.

**Figure 2:**
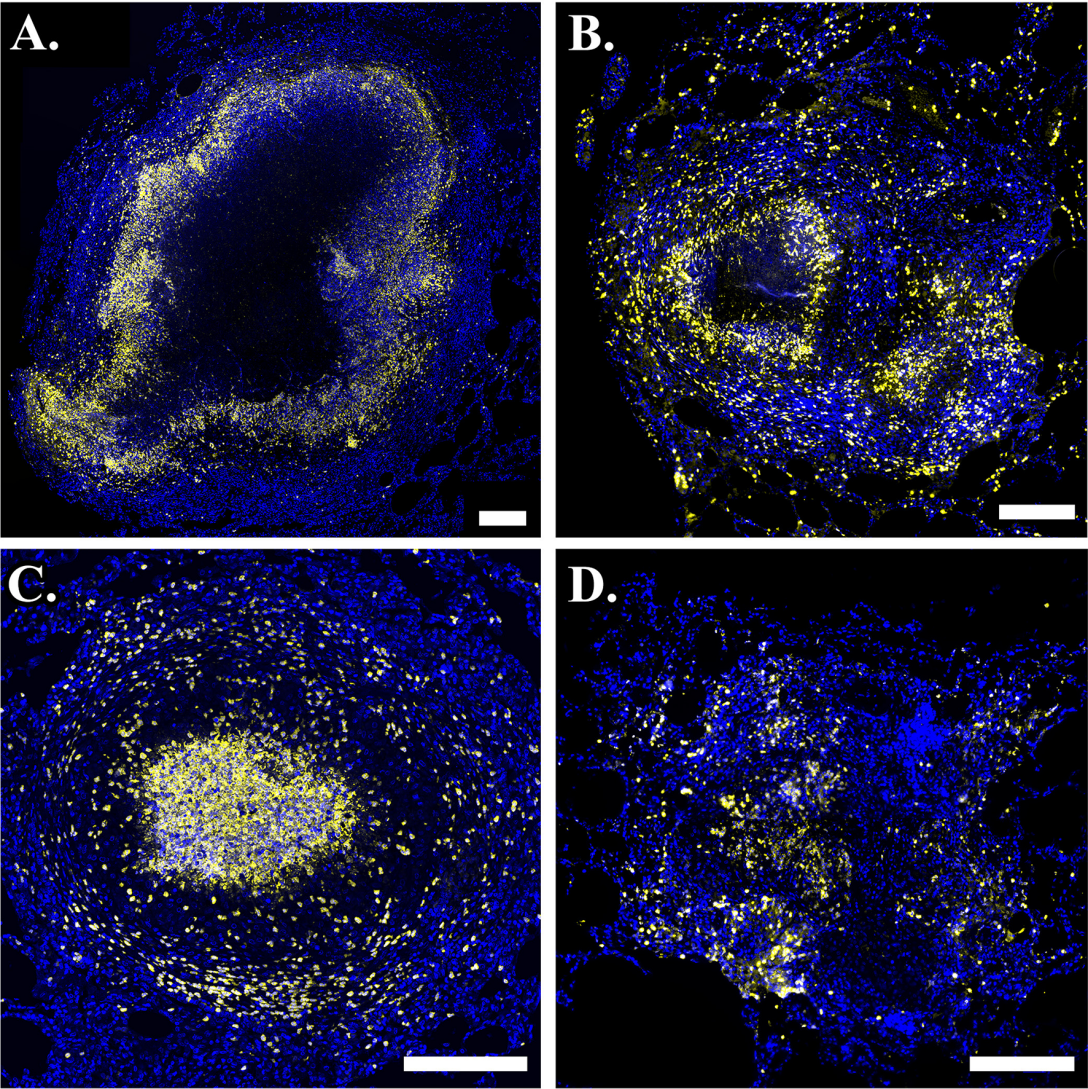
Neutrophils are present in phenotypically-diverse macaque granulomas. Representative granulomas showing **(A)** a necrotic granuloma with a large central region of caseum, **(B)** a granuloma with two necrotic foci on the left and right sides of the granuloma, **(C)** a suppurative granuloma with large numbers of neutrophils infiltrating into the central region, and **(D)** a non-necrotic granuloma. Calprotectin- (S100A9) positive neutrophils are indicated in yellow and DAPI-stained nuclei are indicated in blue. Scale bar represents 250 um.

To ensure that the predictions are robust, we narrowed our parameter space by focusing on only parameters that influence the total number of neutrophils within granulomas for at least 35 consecutive days (5 weeks) and/or parameters that specifically describe neutrophil behavior. Thus, we varied 17 neutrophil-specific parameters and 7 non-neutrophil-specific parameters, as shown in **Table 1**. To determine the values for the 53 remaining previously-varied parameters, we chose a *representative parameter set* from our full parameter set that corresponds to a subset of granulomas we observe experimentally, i.e., a necrotic granuloma with spatial structure similar to that seen in **Figure 2A**, in which a lymphocyte cuff surrounds an epithelioid macrophage layer, an inner neutrophil ring, and a caseous center. We note that doing so merely shifts our region of interest into a more necrotic space, and it does not preclude the formation of other types of granulomas. For this narrow parameter set, we generated 500 granulomas, 5 times each, to produce a set of 2500 total granulomas. From this set, we removed granulomas that did not pass two additional criteria. First, we required that the granuloma diameter remain less than or equal to 3mm throughout the course of the 200-day simulation, as this would ensure that we removed granulomas that grew too large for our computational platform to accommodate. Granuloma size is therefore a limitation of the model; the grid size can be changed to explore larger granulomas, but that was not our focus here. As our second criteria, we required that the total scaled 3D CFU in a granuloma not exceed 10^6^ during the simulation. This is due to the observation that primate granulomas containing CFU greater than 10^6^ are rarely seen in granulomas in experimentally-infected macaques (22, 72), likely due to dissemination or structural changes occurring within granulomas when CFU becomes too high, and our model does not enforce dissemination or account for structural changes in the local lung tissue that may occur in high-burden granulomas. We use this reduced set of 958 granulomas (i.e., 202 granulomas, generated 3-5 times each) for our uncertainty and sensitivity analyses, and will refer to it hereafter as the neutrophil-specific parameter set. In **Table 2**, we provide an overview of the primary granuloma sets generated during the calibration process.

**Table 1:** Parameters that were varied in the neutrophil-specific parameter set.

Parameter definition	Range	Units
Time required for caseation healing	610 —1535	# timesteps
TNF threshold for TNF-induced apoptosis	926 —1366	# internalized TNF bound TNFR1
TNF threshold for NFkB activation	50.8 — 85.6	# molecules
Resiliency of neutrophil to caseum	0.018 — 0.92	n/a
Caseum concentration required to reduce neutrophil lifespan	11 — 50	# caseum-contributing cell deaths
Neutrophil secretion rate of IL10	0.00022 — 0.23 *	# molecules/sec
Neutrophil secretion rate of inactive TGFb	0.00012 — 0.75 *	# molecules/sec
Neutrophil secretion rate of TNF	0.017 — 3 *	# molecules/sec
Neutrophil chemotaxis due to extracellular Mtb	0.066 — 0.96	n/a
Maximum neutrophil lifespan	150 — 780	# timesteps
Maximum number of phagocytosed intracellular Mtb within a neutrophil	5 — 15	# bacteria
Probability that neutrophil death contributes to caseation	0.075 — 0.998	n/a
Probability that neutrophil kills Mtb	0.0023 — 0.20	n/a
Fraction of active TGFb that is bound by a neutrophil	0.0011 — 0.51 *	n/a
Maximum probability to recruit macrophage	0.0010 — 0.028 *	n/a
Chemokine threshold for macrophage and neutrophil recruitment	0.702 — 0.997	# molecules
Minimum probability to recruit a neutrophil	0.0011 — 0.038 *	n/a
Maximum probability to recruit a neutrophil	0.011 — 0.69 *	n/a
Minimum chemokine concentration neutrophils sense during recruitment	0.27 — 9.5	# molecules
Maximum chemokine concentration neutrophils sense during recruitment	128 — 999	# molecules
Neutrophil recruitment due to extracellular Mtb	0.0012 — 0.75	n/a
Neutrophil recruitment due to self-recruitment	0.0011 — 0.24	n/a
Maximum probability to recruit IFN-gamma producing T cell	0.021 — 0.2	n/a
Probability to recruit cognate IFN-gamma producing T cell	0.021 — 0.85 *	n/a

Asterisks denote parameters that were sampled using a log uniform distribution rather than the default uniform distribution. n/a, not applicable.

**Table 2:** Overview of granuloma sets generated in the calibration process.

Name of Granuloma Set	Number of Varied Parameters	Number of Distinct Parameter Sets (i.e., number of different types of granulomas)	Number of Simulation Repetitions Per Parameter Set (i.e., per type of granuloma)	N = Total Number of Granulomas in Set
**Full parameter set**	77	600	3	1800
**Neutrophil-specific parameter set**	24	202	3-5	958

### 2.6 Defining a Spatial Statistic

A better understanding of the spatiotemporal organization of cells in granulomas may shed insight on disease progression, factors contributing to CFU burden, or parameters that can be tuned to make the lung environment more conducive to antibiotic treatment. However, qualitative observation of IHC or simulated images is not a rigorous, user-agnostic method of analysis, nor do such images easily translate to quantitative data that can be used in parameter sensitivity studies. Thus, we define a spatial statistic that enables us to quantitatively evaluate spatial structure by determining the most prevalent type of cell in and around the caseum; namely, the percentage of cells located near the caseum that are neutrophils, macrophages, or T cells. In this work, we use the neutrophil-specific version of the statistic, defined as the number of neutrophils that have at least one fully caseated compartment in their Moore neighborhood divided by the total number of cells that have a least one fully caseated compartment in their Moore neighborhood. This statistic enables us to investigate the parameters that promote the formation of an inner neutrophil ring near the caseous center over time and to thereby hypothesize how the cellular spatial structure of granulomas may relate to different host outcomes.

### 2.7 Virtual Neutrophil Deletion and Depletion Experiments

Virtual neutrophil depletion, i.e., depleting neutrophils at a time *t* post-infection (PI), allows us to understand how the system is affected by the presence or absence of neutrophils. We simulate neutrophil depletion by permanently turning off neutrophil recruitment at time *t*. Neutrophils that are already on the grid at time *t* remain on the grid until they die and are removed from the grid within a few days. We generated four sets of 25 granulomas for depletion at days 25, 50, 75, and 100 PI, respectively, using the same 25 random seeds in each set. Lastly, we set this parameter to the range [day 0, day 200 PI] and used LHS to sample 100 different values at five repetitions each to generate a final dataset. To simulate neutrophil deletion, we turned off neutrophil recruitment for the entirety of the simulation (i.e., no neutrophils ever appear in the simulation).

### 2.8 Quantifying Dissemination

When an infected neutrophil dies, it releases its intracellular bacteria to the local lung environment, where that bacterium remains until it is phagocytosed by another neutrophil or macrophage. An infected neutrophil that breaks away from and dies outside the granuloma could therefore be considered a dissemination event. We define a dissemination statistic as the total number of infected neutrophils that die outside the granuloma boundary. The granuloma boundary is reset at each time step and is defined as in (38), namely, as a circle with center (center of mass x, center of mass y) and radius dictated by the largest distance from the granuloma center to any compartment in the granuloma.

## 3 Results

### 3.1 IHC Staining in Cynomolgus Macaques Illustrates Granuloma Spatial Configurations

Granulomas can manifest in a variety of cellular spatial configurations (73). Experimentally-observed outcomes of infection include clearance with no granuloma formation after infection, clearance of bacteria after granulomas form, and formation of different granuloma types (e.g., necrotic (**Figures 2A, B**), suppurative (**Figure 2C**), non-necrotic (**Figure 2D**) with varying bacteria loads (73, 74). Broadly, necrotic granulomas are granulomas with necrotic (caseous) centers, suppurative granulomas are neutrophil-rich granulomas, particularly in the granuloma center, and non-necrotic granulomas are granulomas that do not contain much necrosis and typically have a cellular center. In this work, we are interested in studying the evolution of granulomas that do not sterilize before the start of the adaptive immune response, as these likely better approximate the responses of individuals who develop active disease and ultimately seek treatment, as well as those that do sterilize before the start of the adaptive response, as doing so may help elucidate mechanisms of successful control.

We use immunohistochemistry (IHC) to study patterns of neutrophil localization in NHPs. In **Figure 2**, we show images of granulomas from different NHPs with active TB. Images A-D serve as sample granulomas in the model calibration and validation process, in which we required *GranSim* to produce a range of experimentally-observed outcomes. In images A and B, neutrophils (stained yellow) form an inner ring next to the caseum and sparsely populate the lymphocyte cuff. Both of these images are examples of typical necrotic granulomas. In image B, there appears to be a second concentrated region of neutrophils to the lower right of the caseous center. This suggests that there are two necrotic foci in the granuloma and that a second caseous center is forming, perhaps because the cells in that location that are controlling the infection poorly. Image C represents a suppurative granuloma where neutrophils (yellow) are present throughout the granuloma but are enriched in the granuloma’s center. Image D shows a non-necrotic granuloma characterized by lack of caseum where neutrophils are not concentrated into distinct foci. This image highlights the complexity of granuloma structure and response to infection, as well as some inherent challenges in IHC image analysis.

### 3.2 Computational Model, GranSim, Captures Neutrophil Spatiotemporal Organization and Dynamics Within TB Granulomas

**Figure 3:**
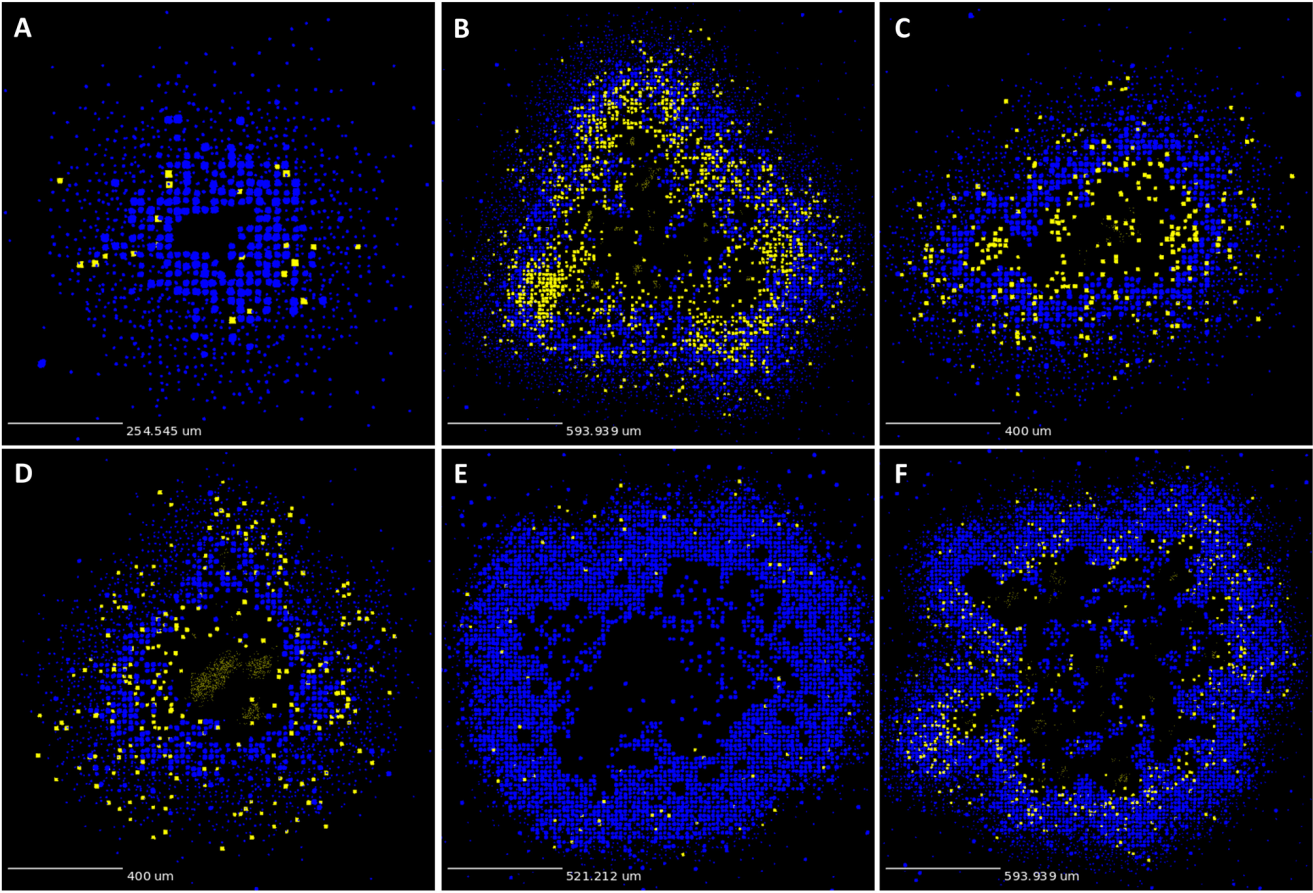
*GranSim* produces simulated granulomas that reflect granulomas in experimentally-infected macaques. Panels **(A–F)** show six sample simulated granulomas drawn from the full parameter set (see **Table 2** and **Online Supplement Table 1**), shown at 200 days post-infection. Neutrophils are indicated in yellow and all other cell types are indicated in blue, in order to correlate with the color scheme used for the IHC images in **Figure 2**. Extracellular Mtb visualized as pale yellow patches. Caseum not shown.

**Figure 4:**
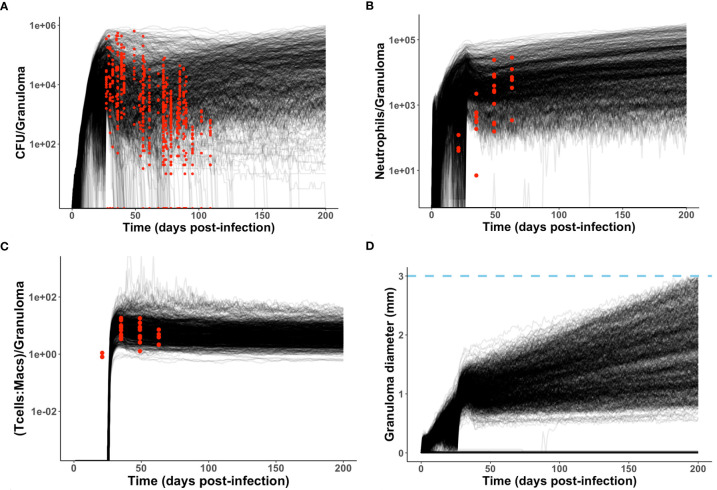
Model produces simulated data that matches NHP data. We compare simulated data from the neutrophil-specific parameter set with experimental datasets obtained from (34) (see Methods). Red = experimental data, black = simulated data. **(A, C, D)** Number of bacteria (CFU) and T cell to macrophage cell count ratio were the primary temporal experimental data used in model calibration; granuloma diameter (in mm) served as an additional screening tool. **(B)** Neutrophil cell count data was used for model validation.

As described in Methods, we use *GranSim* to create a repository of 958 simulated granulomas. In **Figure 3**, we present six different simulated granulomas from this repository, showing that our model produces spatial organizations that qualitatively match NHP IHC images, such as those in **Figure 2**. These granulomas are achieved through parameter variation that we discuss further below. In particular, these simulated granulomas are all necrotic granulomas and have a distinct caseous center, and in panels 3B, C, and D neutrophils are primarily found either in an inner ring closest to the caseum or in the lymphocyte cuff. In time-lapse videos for **Figure 3** (**Videos 3A–F**), we visualize the full-color scheme for the six granulomas from **Figure 3** (at http://malthus.micro.med.umich.edu/lab/movies/neutrophil/). In **Figure 4**, we show that the temporal CFU, neutrophil cell count, and T cell to macrophage cell count ratio qualitatively and quantitatively match the trends observed in the NHP studies [e.g., see (34)]. Beyond matching to experimental CFU levels, cell ratios, and spatial structures, our model recapitulates several additional key outcomes that have been observed experimentally regarding within-host heterogeneity (22).

### 3.3 Parameters Associated With Neutrophil Behavior Drive CFU, Neutrophil Counts, T Cell to Macrophage Ratios, and Granuloma Spatial Organization

To test whether neutrophils play an important role in the progression of TB, we performed sensitivity analyses to determine the primary mechanisms driving granuloma outcomes. A major advantage of *GranSim* is the ability to simulate and analyze granuloma formation over time, and thus we calculated PRCC values across each of the 200 days PI. Using our neutrophil-specific parameter set (as defined in Methods), we sought to identify which parameters have persistent, significant effects on these outcomes over time.

In **Table 3**, we list parameters that we predicted to have significant PRCC values for an extended period of time during the first 200 days PI (p <0.001). We identify model parameters that significantly influence total CFU, neutrophil cell count, T cell to macrophage ratio, and granuloma spatial structure (**Table 3**). For example, reducing neutrophil lifespan or increasing the rate of secretion of TNF by neutrophils each correspond to a reduction in total CFU/granuloma. Interestingly, many parameters that significantly affect both CFU and neutrophil counts share the same correlation sign. This suggests that bacterial growth and neutrophil accumulation may be driven by some of the same biological mechanisms. Therefore, at least when considered over time periods of several weeks to months, this finding supports the observation that higher neutrophil numbers are associated with poorer granuloma outcomes.

**Table 3:** PRCC analysis of the neutrophil-specific parameter set, with respect to CFU, neutrophil count, T cell to macrophage ratio, and spatial structure.

Parameter definition	CFU	Neutrophil count	Tcell/Mac count ratio	Spatial structure
TNF threshold for TNF-induced apoptosis	Positive	Positive		
TNF threshold for NFkB activation	Positive		Positive	
Resiliency of neutrophil to caseum				Positive
Caseum concentration required to reduce neutrophil lifespan	Positive		Positive	Positive
Neutrophil secretion rate of inactive TGFb				Negative
Neutrophil secretion rate of TNF	Negative	Negative	Negative	
Neutrophil chemotaxis due to extracellular Mtb	Negative		Negative	Negative
Maximum neutrophil lifespan	Positive	Positive	Negative	Positive
Probability that neutrophil death contributes to caseation	Negative	Positive	Negative	Negative
Probability that neutrophil kills Mtb	Negative		Negative	Negative
Fraction of active TGFb that is bound by a neutrophil			Negative	Positive
Maximum probability to recruit macrophage	Positive	Positive	Negative	Positive
Neutrophil recruitment due to extracellular Mtb				Negative
Maximum probability to recruit a neutrophil		Positive	Negative	Positive
Maximum chemokine concentration neutrophils sense during recruitment			Positive	Negative
Minimum probability to recruit a neutrophil	Negative	Positive	Negative	Positive
Minimum chemokine concentration neutrophils sense during recruitment			Negative	
Maximum probability to recruit IFN-gamma producing T cell	Negative	Negative	Positive	Negative
Probability to recruit cognate IFN-gamma producing T cell	Negative		Negative	Negative

Shown are parameters that are significant for at least 35 consecutive days during the 200-day span (p**<**0.001). If the correlation sign was both positive and negative during the 200 days, then each sign was considered separately for the 35-day requirement. See Methods for a description of the spatial structure statistic.

However, not all parameters that drive CFU also drive neutrophil count, and vice versa. This suggests that lung micro-environments that permit a higher rate of neutrophil recruitment could support granulomas with high neutrophil counts and low CFU. In contrast, other lung micro-environments could support granulomas with relatively few neutrophils but higher bacterial loads, such as those where the TNF threshold for NFkB activation is higher, the caseum levels required to shorten local neutrophil lifespan are greater, extracellular Mtb plays a smaller role in determining neutrophil directional movement, or neutrophils have reduced capacity to kill Mtb. We also note the significance of the parameter “probability that neutrophil death contributes to caseation”, which defines the likelihood that a neutrophil death will contribute to caseum rather than be phagocytosed by a macrophage. Our analysis suggests that increased caseum levels due to neutrophil death also correlate with lower CFU. This suggests that caseum may have a beneficial purpose, perhaps through trapping and physically constraining Mtb. Much work by Dannenberg in the last century supports the idea that caseum has an overall beneficial effect on control (75).

The spatial statistic shown in the final column of **Table 3** enables us to better understand the arrangement of cells within simulated granulomas. Greater values of this statistic correspond to granulomas with higher percentages of neutrophils (as opposed to other cell types) composing the population of cells located in or near caseum. These are likely granulomas with more defined inner neutrophil rings. Interestingly, we see that parameters that are positively or negatively correlated with CFU are often also positively or negatively correlated with this spatial statistic as well, perhaps suggesting that the presence of neutrophils in or near caseum is a hallmark of high CFU granulomas.

### 3.4 Neutrophils Influence Mtb Levels During Both Innate and Adaptive Immunity

Although neutrophils are typically associated with innate immunity and acute inflammation, recent work suggests that they also play roles in chronic inflammatory conditions and adaptive immune responses (3, 40). Neutrophil dynamics within granulomas during early stages of Mtb infection can have long-lasting ramifications on disease progression (4). Using the neutrophil-specific parameter set and corresponding uncertainty and sensitivity analyses, we investigated which neutrophil-associated parameters directly or indirectly influenced CFU levels during either the innate or adaptive immune responses. Importantly, we found that the biological mechanisms driving CFU levels differ in relative importance and contribution over time.

We identified neutrophil-specific parameters that are significantly correlated with CFU at six different time points PI (**Table 4**) and noted if this significance occurs during the innate (days 7, 14, 21 PI) or adaptive (days 28, 70, 105 PI) immunity-driven stages of TB, respectively (p <0.001). We report the correlation (PRCC) values for each significant parameter, and we consider significant parameters at each time point in order of descending PRCC value magnitude (as they are likely to be the strongest correlated). We found that neutrophilic effects on CFU are not solely limited to the innate immunity-dominated stage of disease but are also evident during adaptive immunity (**Table 4**). In order to directly compare PRCCs, we use a Fishers Z test (65). For each of the three time points within the innate immune response, neutrophil secretion of TNF has the strongest correlation with CFU (**Table 4**). This suggests that promoting neutrophil secretion of TNF early in infection may help control CFU growth; that neutrophil secretion of TNF remains a significant (although less influential) parameter during the adaptive response suggests that this benefit persists during the adaptive response. The tables corresponding to days 28, 70, and 105 PI (during adaptive response) identify the eight, four, and three parameters, respectively, that directly represent neutrophil behavior and significantly affect total CFU. At day 28 PI, the maximum lifespan of a neutrophil and the likelihood that a dead neutrophil contributes to caseum levels have the strongest influence on CFU, after neutrophil secretion of TNF. This suggests that neutrophil longevity and interactions with caseum are more influential in governing total CFU than parameters that tune neutrophil chemotaxis or bactericidal effects. As the time point at 28 days PI is just after both the onset of the adaptive response in the simulations and the typical experimentally-observed peak in CFU (see **Figure 4A**), we further note that these parameters (and by association, neutrophils) likely play a key role in determining the severity/magnitude of that CFU peak (**Table 4**). By days 70 and 105 PI, the Fishers Z test identifies the most influential parameter on CFU to be a macrophage recruitment parameter, suggesting that, although neutrophilic effects on CFU persist into the adaptive response stage, neutrophils may no longer be the primary driver of CFU.

**Table 4:** Innate *versus* adaptive factors driving infection.

Varied Parameters (Innate Response)	Day 7 PI	Day 14 PI	Day 21 PI
Neutrophil secretion rate of TNF	-0.491*	-0.578*	-0.489*
Maximum neutrophil lifespan		0.474*	0.485*
Probability that neutrophil kills Mtb	-0.234*	-0.358*	-0.205
Probability that neutrophil death contributes to caseation		-0.289	-0.396*
Minimum probability to recruit a neutrophil		-0.288	-0.221
Maximum probability to recruit macrophage	0.200*	0.240	0.283
Neutrophil chemotaxis due to extracellular Mtb		-0.182	-0.161
Caseum concentration required to reduce neutrophil lifespan		0.152	0.329
Maximum number of phagocytosed intracellular Mtb within a neutrophil			0.168
TNF threshold for TNF-induced apoptosis			0.148
TNF threshold for NFkB activation			0.134
**Varied Parameters (Adaptive Response)**	**Day 28 PI**	**Day 70 PI**	**Day 105 PI**
Maximum probability to recruit macrophage	0.336	0.4426*	0.497*
Neutrophil secretion rate of TNF	-0.529*	-0.4425*	-0.452*
Probability to recruit cognate IFN-gamma producing T cell		-0.387*	-0.441*
Maximum neutrophil lifespan	0.427*		
Probability that neutrophil death contributes to caseation	-0.396*		
TNF threshold for NFkB activation	0.151	0.199	0.198
Maximum probability to recruit IFN-gamma producing T cell		-0.162	-0.191
Probability that neutrophil kills Mtb	-0.228	-0.158	-0.185
Caseum concentration required to reduce neutrophil lifespan	0.351	0.153	
Neutrophil chemotaxis due to extracellular Mtb	-0.184	-0.149	-0.144
Minimum probability to recruit a neutrophil	-0.300		
TNF threshold for TNF-induced apoptosis			0.136
Maximum number of phagocytosed intracellular Mtb within a neutrophil	0.135		

Here we show three selected time points chosen during the innate immune response time frame, and three selected time points chosen during the adaptive immune response time frame. Only significant correlations are shown (p **<**0.001), and varied parameters that do not significantly influence CFU for at least one of these six time points are not listed here. We identify the three parameters with the highest PRCC value magnitude for each day with asterisks. We then used Fishers Z test to directly compare PRCCs between these parameters.

That neutrophils can influence CFU levels later in infection (e.g., days 70, 105 PI) suggests that they may play an important role outside of the innate immune response, and thus may prove a useful target for drug therapy. Here, we suggest that the positive or negative nature of neutrophilic influence is not clear-cut, as heterogeneity in the neutrophil population – represented here *via* parameter ranges – may modulate the severity of host responses. This result is not contradictory to evidence showing that neutrophils can be harmful later in TB; rather, it suggests that although neutrophil behaviors can tune CFU levels, neutrophils may fail to accumulate in large enough numbers to have observable beneficial effects, exhibit antagonistic positive and negative behaviors, or contribute to a spectrum of poor granuloma outcomes.

### 3.5 Virtual Neutrophil Depletion in Granulomas with High Bacterial Burdens Reduces CFU

**Figure 5:**
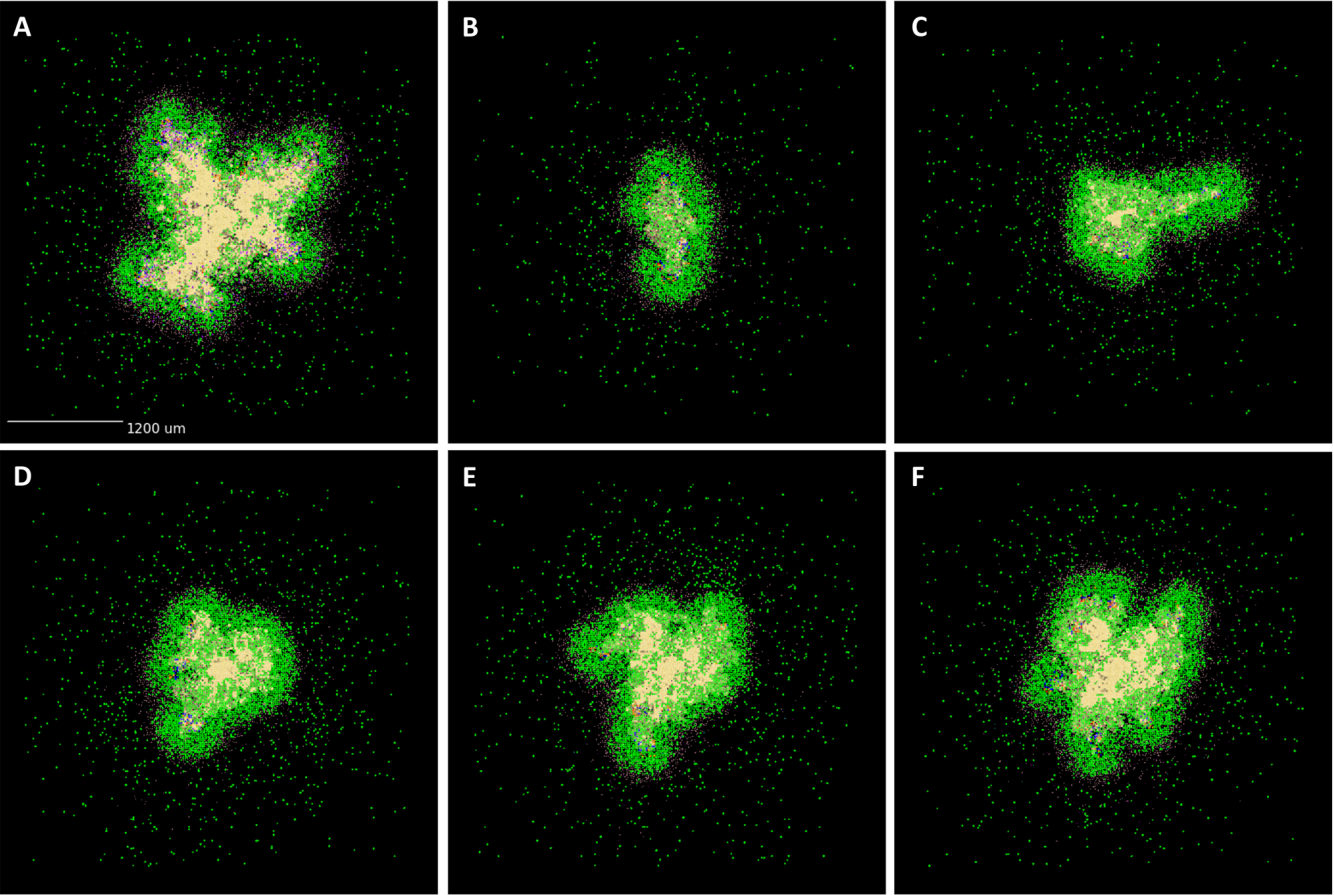
Spatial outcomes for virtual neutrophil deletion and depletion studies. Simulated granulomas at day 200 PI. Macrophages (green– resting, orange– infected, red– chronically infected, blue– activated), Neutrophils (purple), T cells (Tgam– pink, Tcyt– maroon, Treg– cyan), extracellular Mtb (brown), and caseum (beige) are shown. **(A)** As a wild-type control, we use a simulated granuloma that has high levels of CFU and abundant levels of neutrophils at 200 days PI. **(B–F)** are the virtual deletion and depletions using this same granuloma set. **(B)** Neutrophil deletion. Neutrophils are not present on the grid at any point during the simulation. **(C–F)** Virtual neutrophil depletion at days 25, 50, 75, and 100 PI, respectively.

**Figure 6:**
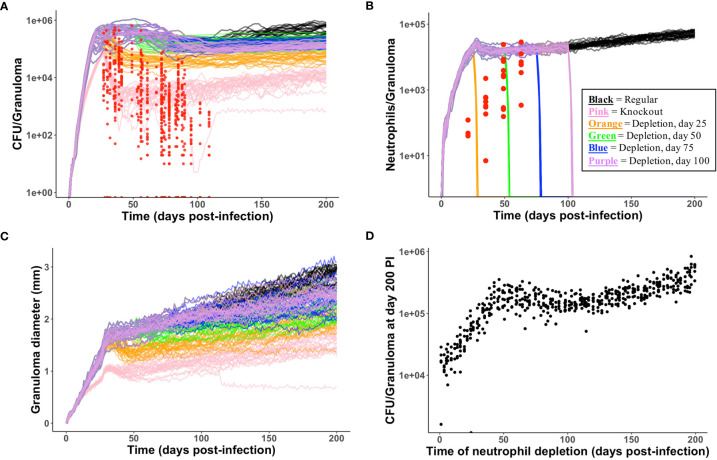
Temporal outcomes for virtual neutrophil deletion and depletion studies. **(A–C)** show temporal simulated data for the six virtual experiments (control, neutrophil deletion, and four temporally-distinct neutrophil depletions). Each simulated data set contains 25 granulomas, one of which is shown in **Figure 5**. The color key is shown in **(B). (A)** CFU versus days PI. Red = experimental data from NHPs. We observe that deleting or depleting neutrophils reduces total CFU at day 200. **(B)** Neutrophil cell counts versus days PI are shown. Neutrophil count quickly goes to zero following neutrophil depletion. **(C)** We observe that deleting or depleting neutrophils reduces granuloma diameters at day 200. **(D)** Using the control parameter set, we varied the time PI of neutrophil depletion. At day 200, reduced CFU correlates with earlier neutrophil depletion PI.

High neutrophil levels within TB lung granulomas are associated with poor host outcomes (60, 76–80). One benefit to using *GranSim* is that we can repeatedly simulate the same granuloma under different conditions. Here, we investigate the effect of virtually depleting or deleting neutrophils from granulomas that have poor outcomes, defined here as high CFU. From our neutrophil-specific parameter set (as defined in Methods), we chose a parameter set that consistently produced granulomas with high CFU (greater than 10^5^) and high neutrophil cell counts (greater than 10^4^) at day 200 PI (see **Figure 5** panel A for the control case). We used this parameter set to perform a virtual deletion and depletion of neutrophils at various days PI. Using the same 25 random seeds for each simulation, we generated six sets of 25 granulomas: control granulomas, neutrophil deletion granulomas (i.e., granulomas in which no neutrophils are present at the start of or during the simulation), and neutrophil depletion granulomas, for which neutrophil depletions were imposed at day 25, 50, 75, or 100 PI. In **Figure 6**, we show that deleting or depleting neutrophils from this subtype of granuloma results in a lower CFU by day 200 PI than that of the control. Qualitatively, we observe that the earlier post-infection neutrophils are eliminated, the lower the total CFU at day 200 PI and the smaller in diameter the granuloma. Neutrophil deletion produces the lowest total CFU at day 200 PI and a lower early CFU peak. Similarly, virtual depletion of neutrophils at day 25 PI reduces the early CFU peak and produces the next lowest total CFU at day 200 PI. The size and structure of these granulomas shown in **Figure 5**, where we show a sample granuloma for each of the six virtual experiments at day 200 PI, further reflect these qualitative trends.

We performed these same six virtual experiments on two additional granuloma parameter sets whose control granulomas produced high CFU and high neutrophil count late in infection, and saw similar qualitative behavior in which neutrophil deletion and early depletion led to reduced CFU and granuloma size (data not shown). We hypothesize that, in the case of granulomas with high CFU burdens and high neutrophil count, neutrophils are a key driver of, and serve to amplify, uncontrolled bacterial growth and poor outcomes.

Interestingly, when we performed virtual neutrophil knockout and depletion experiments using a parameter set that corresponded to granulomas that effectively control CFU growth, we did not observe the same results; most notably, a neutrophil deletion or depletion at day 25 PI resulted in CFU levels at day 200 that were ∼2-2.5 orders of magnitude higher than those of the control granulomas, as shown in **Online Supplement Figure 2** and **Online Supplement Figure 3**. Furthermore, when we performed neutrophil knockout and depletion experiments using a parameter set that corresponded to granulomas with low, controlled CFU at day 200 PI (e.g., 0 ≤ CFU < ∼10^3^), we observed an even stronger relationship between neutrophil deletion/depletion and uncontrolled CFU growth. As shown in **Online Supplement Figure 4** and **Online Supplement Figure 5**, the earlier that neutrophils are removed from the local lung environment, the worse the outcome, as both CFU and granuloma size increase. This suggests that neutrophils may play an important role in controlling bacterial growth in some granulomas. This may also account for some of the variability reported in the literature (5).

### 3.6 Neutrophils Can Facilitate Local Dissemination and Granuloma Budding

Dissemination is associated with the development of active TB and accelerated disease progression (81). It occurs when a granuloma fails to control and physically contain bacterial growth, resulting in infected cells escaping the physical boundaries of the granuloma and Mtb spreading beyond the local lung environment. Recent work by Wessler et al. (2020) investigates how dissemination appears, as well as the potential role of multifunctional CD8+ T cells and macrophage dynamics (34). The literature also suggests a potential link between neutrophils and dissemination (5, 8, 10, 82); thus, here we investigate the mechanisms driving dissemination, focusing on the role of neutrophils.

**Figure 7:**
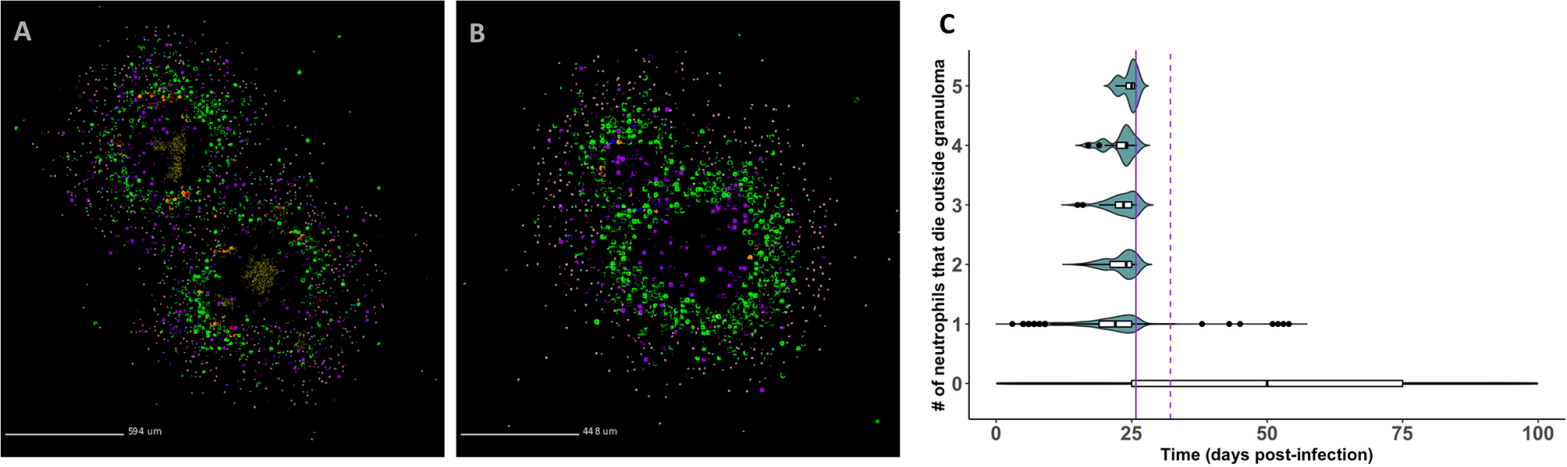
*GranSim* predicts that neutrophils contribute to mycobacterial dissemination and granuloma budding. **(A)** A granuloma that has disseminated, resulting in two granulomas by day 200 PI. **(B)** A granuloma with budding, also shown at day 200 PI. We identified granulomas in panels **(A, B)** from the full parameter set (see **Table 2**). Macrophages (green– resting, orange– infected, red– chronically infected, blue– activated), Neutrophils (purple), T cells (Tgam–pink, Tcyt– maroon, Treg– cyan), extracellular Mtb (brown). Caseum is not shown in order to better visualize spatial locations of different cell types. **(C)** Frequency of neutrophil-facilitated dissemination events over time. We visualize data for all runs in the neutrophil-specific parameter set. The purple vertical lines denote the start of the adaptive response (solid = T cell recruitment begins, dotted = T cell recruitment is fully ramped up).

In **Figures 7A, B**, we show that infected neutrophils can facilitate local dissemination and budding, respectively. Notably, these events occur as emergent phenomena in our model, arising in a subset of the replications generated for each granuloma due to the stochasticity present in the system. In **Figure 7A**, we show an example of dissemination. Of the five replications generated for this granuloma and tracked for 200 days PI, a single granuloma formed in three of the replications, a disseminating granuloma formed in one of the replications (shown here), and no granuloma formed in one of the replications due to the host clearing the bacteria early after infection. In **Videos 7A–C** (at http://malthus.micro.med.umich.edu/lab/movies/neutrophil/), we provide time-lapse videos for the granuloma shown in **Figure 7A**. We show that following the formation of a single granuloma, an infected neutrophil breaks away from the original granuloma within the first week post-infection. After traveling to a distal part of the lung micro-environment, where it dies and releases its bacteria around day 7 PI, a resting macrophage phagocytoses the extracellular Mtb by day 20 PI, instigating the formation of a second granuloma. We distinguish this from budding, where a granuloma pinches off a new granuloma (**Figure 7B**).

To quantify the frequency of neutrophil-facilitated dissemination events and determine the mechanisms driving them, we create a new dissemination statistic. Described in Methods, this statistic measures the frequency with which an infected neutrophil “escapes” and dies outside the granuloma. In **Figure 7C**, we show that these events almost exclusively occur during the innate immune response timeframe. This suggests that neutrophils contribute to local dissemination during the innate immunity-driven response, and perhaps are able to “seed” new disseminating granulomas before the adaptive immunity-driven response kicks in. The sharp decline in frequency of these events following the start of the adaptive response suggests that T cells play an important role in limiting neutrophil-facilitated dissemination. We used PRCC analyses to identify factors driving dissemination. We can decrease total numbers of infected neutrophils that die outside a granuloma, and thereby decrease potential neutrophil-facilitated dissemination events, by modulating several parameters. We can affect these changes by: increasing the lifespan of neutrophils, decreasing the proportion of neutrophils that contribute to local caseum at death, increasing the likelihood of macrophage recruitment (p < 0.001). From this, we hypothesize that micro-environmental conditions that contribute to early neutrophil death could increase the frequency of dissemination events, whereas efferocytosis or perhaps just higher macrophage numbers may decrease the frequency of dissemination events.

## 4 Discussion

Gaining a better understanding of the immune response to Mtb is crucial to addressing increased prevalence of multi-drug resistant strains, the current complexity and length of treatment, and the inherent difficulties of experimental work. In this work, we extended our hybrid agent-based computational model, *GranSim*, to include neutrophils as an explicit cell type. We were particularly interested in investigating mechanistic bases for how and why neutrophils play dual roles in immunity to Mtb, where in some settings they have beneficial roles and in others appear to have detrimental effects (5, 13, 14, 83).

Several of our results address this dichotomous perspective of neutrophil contribution to pathology. In our depletion studies, we observed that neutrophils contribute to bacterial growth in some granulomas but help control bacterial growth in others. Interestingly, neutrophils seemed to amplify granuloma outcomes, as depleting neutrophils from granulomas with high CFU burdens reduced the bacterial burden in those granulomas (suggesting they are making a bad situation worse), while depleting neutrophils from granulomas with low bacterial burdens increased the number of bacteria per granuloma (suggesting that they are a stabilizing factor in these granulomas). The results of these studies suggest that the degree to which neutrophils contribute to pathology may vary along a spectrum at the individual granuloma level. Potential neutrophil-directed therapies will therefore likely require a nuanced approach, as simply eliminating all neutrophils in the lung environment could impair immunity in some granulomas while promoting anti-mycobacterial responses in others. This also highlights an advantage of computational modeling over *in vivo* experiments: we can generate a large set of heterogeneous granulomas to analyze at a single-granuloma level. Through consideration of different parameter sets, this enables us to identify local niches of the lung conducive to CFU growth that might be missed when averaging across the whole lung environment (62).

Similarly, our uncertainty and sensitivity analyses suggest that neutrophils can both promote and reduce CFU growth. We predict that neutrophil behavior influences CFU levels in granulomas and that some of the same biological mechanisms may underlie both bacterial growth and neutrophil accumulation. By identifying neutrophil-specific biological mechanisms that drive CFU, such as reduced neutrophil TNF secretion, we link neutrophils with poor outcomes. However, as in the case of the neutrophil depletion studies, high neutrophil count is not exclusively associated with high CFU, nor is low neutrophil cell count necessarily correlated with low CFU.

*GranSim* captures apoptosis and necrosis, but other forms of cell death are increasingly being viewed as important modulators of immunity, such as death through neutrophil NETosis and macrophage phagocytosis of an apoptotic neutrophil (efferocytosis) (84). For the purposes of this study, we do not simulate neutrophil extracellular traps (NETs) but there is data suggesting that NETs cannot eliminate Mtb (85). As more data on NETs in human and NHP TB become available, particularly with regard to the role of NETs in innate immunity (86), it will become worthwhile to model them in a future study. Similarly, a neutrophil’s ability engage in tissue-remodeling activities through MMPs has implications for granuloma function, but at the present time, we lack a sufficient amount of data on temporal MMP expression and the relationship between MMP expression, bacterial burden, and granuloma phenotypes to accurately model these enzymes in *GranSim*. Efferocytosis has been observed in NHP granulomas (21) and we indirectly account for this behavior through a probability parameter associated with caseum levels. By linking this parameter to the proximity of a dying neutrophil and a macrophage, we could more directly investigate this relationship. There are many future avenues of interest we can pursue: the relationship between CFU and neutrophil proximity to caseum, identification of parameters associated with specific granuloma spatial structures, the potential role of neutrophils in creating a growth-permissive environment, etc. Mishra et al. showed in mice that PMNs may be a nutrient reservoir for Mtb (78); if Mtb in NHP granulomas are primarily located in or near caseous environments, perhaps neutrophils are the reason why. We are also interested in the relationship between neutrophils and T cells over the course of infection. For example, Gideon et al. (2019) showed that peripheral blood neutrophils can suppress Mtb-specific T cell responses *in vitro* but did not conclusively identify a relationship between neutrophils and T cell cytokine expression *in vitro* (21). When we have stronger data on this interaction in NHP granulomas, we can update *GranSim* to include these cell-cell interactions. Pairing of wet lab studies together with modeling will further elucidate the role of neutrophils in TB.

In NHPs, Mtb infection is often not detectable during the innate immunity-dominated phase of TB, as visible granulomas are not detected by PET/CT imaging or histopathologic examination until 3–4 weeks post-infection (23, 87). This suggests that the adaptive response will already be underway by the time treatment commences. Thus, therapies need to target cell behavior specific to cells in the adaptive response, or, in the case of granulomas that newly form during the adaptive immunity-dominated stage of disease, cell behavior specific to both the innate and adaptive immunity-driven responses. Our study also highlights the value of *GranSim* as a tool for studying neutrophil dynamics and neutrophil-regulated immunity through the full course of TB from the point of initial infection to established or resolved disease.

Past work has identified multiple neutrophil waves during the immune response to Mtb (8, 14, 18, 88) and suggested that early and late neutrophil recruitment may play distinct roles in infection (18). In particular, recent work has begun to consider the role of neutrophils beyond the innate response and as modulators of adaptive immunity (3, 40, 83, 89). We investigated the distinct role of neutrophils during both innate and adaptive immunity, with a particular focus on the adaptive response. Through uncertainty and sensitivity analyses, we found that neutrophils directly modulate bacterial levels during the adaptive response, and that mechanisms such as neutrophil longevity and interactions with caseum may be useful targets for therapies. Consideration of neutrophil heterogeneity, both phenotypically and functionally, in future work with *GranSim* could further elucidate this process (43, 76, 90).

We identified some of these same mechanisms as drivers of neutrophil-facilitated local dissemination. Notably, we showed that these types of dissemination events primarily occur during the innate immunity-driven stage of infection. That the frequency of these events abruptly drops off at the onset of the adaptive immune response highlights the importance of T cells to effective physical containment of bacterial growth as well as the potential of neutrophils to influence granuloma shape and severity. This suggests neutrophils as a potential mechanism through which Mtb can exploit its granuloma environment during innate immunity for dissemination (91).

## Data Availability Statement

The raw data supporting the conclusions of this article will be made available by the authors, without undue reservation.

## Ethics Statement

All animal protocols and procedures were approved by the University of Pittsburgh’s Institutional Animal Care and Use Committee (IACUC) that adheres to the national guidelines established in the Animal Welfare Act and Guide for the Care and Use of Laboratory Animals as mandated by the U. S. Public Health Service Policy (PHS).

## Author Contributions

CH performed the modeling studies and drafted the manuscript. JTM and HPG provided the NHP IHC and cell number data, respectively. DEK, JJL, and JTM oversaw the project. All authors edited the manuscript and approved the final submitted version.

## Funding

The work in this paper was funded under NIH grants R01AI123093 and R01HL110811 awarded to DEK and JJL. The simulations used the Extreme Science and Engineering Discovery Environment (XSEDE) computing cluster, which is supported by National Science Foundation grant ACI-1548562.

## Conflict of Interest

The authors declare that the research was conducted in the absence of any commercial or financial relationships that could be construed as a potential conflict of interest.

## Publisher’s Note

All claims expressed in this article are solely those of the authors and do not necessarily represent those of their affiliated organizations, or those of the publisher, the editors and the reviewers. Any product that may be evaluated in this article, or claim that may be made by its manufacturer, is not guaranteed or endorsed by the publisher.
